# Protocol for the 2ND-STEP study, Japan Clinical Oncology Group study JCOG1802: a randomized phase II trial of second-line treatment for advanced soft tissue sarcoma comparing trabectedin, eribulin and pazopanib

**DOI:** 10.1186/s12885-023-10693-w

**Published:** 2023-03-08

**Authors:** Makoto Endo, Tomoko Kataoka, Toshifumi Fujiwara, Satoshi Tsukushi, Masanobu Takahashi, Eisuke Kobayashi, Yoko Yamada, Takaaki Tanaka, Yutaka Nezu, Hiroaki Hiraga, Junji Wasa, Akihito Nagano, Kenji Nakano, Robert Nakayama, Tetsuya Hamada, Masanori Kawano, Tomoaki Torigoe, Akio Sakamoto, Kunihiro Asanuma, Takeshi Morii, Ryunosuke Machida, Yuta Sekino, Haruhiko Fukuda, Yoshinao Oda, Toshifumi Ozaki, Kazuhiro Tanaka

**Affiliations:** 1 Department of Orthopaedic Surgery, Graduate School of Medical Sciences, Kyushu University, Fukuoka, Japan; 2 JCOG Data Center/Operations Office, National Cancer Center Hospital, Tokyo, Japan; 3 Department of Orthopaedic Surgery, Aichi Cancer Center, Nagoya, Japan; 4 Department of Clinical Oncology, Tohoku University Graduate School of Medicine, Sendai, Japan; 5 Department of Musculoskeletal Oncology, National Cancer Center Hospital, Tokyo, Japan; 6 Department of Breast and Medical Oncology, Saitama Cancer Center, Saitama, Japan; 7 Department of Orthopaedic Surgery, Fukui University School of Medicine, Fukui, Japan; 8 Department of Musculoskeletal Tumor Surgery, Kanagawa Cancer Center, Yokohama, Japan; 9 Department of Musculoskeletal Oncology, National Hospital Organization Hokkaido Cancer Center, Sapporo, Japan; 10 Division of Orthopaedic Oncology, Shizuoka Cancer Center, Nagaizumi, Japan; 11 Department of Orthopaedic Surgery, Gifu University School of Medicine, Gifu, Japan; 12 Department of Medical Oncology, Cancer Institute Hospital of the Japanese Foundation for Cancer Research, Tokyo, Japan; 13 Department of Orthopaedic Surgery, Keio University School of Medicine, Tokyo, Japan; 14 Department of Orthopaedic Surgery, Kurume University School of Medicine, Kurume, Japan; 15 Department of Orthopaedic Surgery, Faculty of Medicine, Oita University, 1-1 Idaigaoka, Yufu City Oita, Hasama 879-5593 Japan; 16 Department of Orthopaedic Oncology and Surgery, Saitama Medical University International Medical Center, Hidaka, Japan; 17 Department of Orthopaedic Surgery, Kyoto University Graduate School of Medicine, Kyoto, Japan; 18 Department of Orthopaedic Surgery, Mie University Graduate School of Medicine, Tsu, Japan; 19 Department of Orthopaedic Surgery, Faculty of Medicine, Kyorin University, Mitaka, Japan; 20 Department of Anatomic Pathology, Graduate School of Medical Sciences, Kyushu University, Fukuoka, Japan; 21 Department of Orthopaedic Surgery, Science of Functional Recovery and Reconstruction, Faculty of Medicine, Dentistry, and Pharmaceutical Sciences, Okayama University, Okayama, Japan; 22 Department of Advanced Medical Sciences, Oita University, 1-1 Idaigaoka, Yufu City Oita, Hasama 879-5593 Japan

**Keywords:** Soft tissue sarcoma, Chemotherapy, Metastatic, Unresectable, Advanced stage, Second-line, Trabectedin, Eribulin, Pazopanib, Randomized phase II trial

## Abstract

**Background:**

Soft tissue sarcomas (STS) are a rare type of malignancy comprising a variety of histological diagnoses. Chemotherapy constitutes the standard treatment for advanced STS. Doxorubicin-based regimens, which include the administration of doxorubicin alone or in combination with ifosfamide or dacarbazine, are widely accepted as first-line chemotherapy for advanced STS. Trabectedin, eribulin, pazopanib, and gemcitabine plus docetaxel (GD), which is the empirical standard therapy in Japan, are major candidates for second-line chemotherapy for advanced STS, although clear evidence of the superiority of any one regimen is lacking. The Bone and Soft Tissue Tumor Study Group of the Japan Clinical Oncology Group (JCOG) conducts this trial to select the most promising regimen among trabectedin, eribulin, and pazopanib for comparison with GD as the test arm regimen in a future phase III trial of second-line treatment for patients with advanced STS.

**Methods:**

The JCOG1802 study is a multicenter, selection design, randomized phase II trial comparing trabectedin (1.2 mg/m^2^ intravenously, every 3 weeks), eribulin (1.4 mg/m^2^ intravenously, days 1 and 8, every 3 weeks), and pazopanib (800 mg orally, every day) in patients with unresectable or metastatic STS refractory to doxorubicin-based first-line chemotherapy. The principal eligibility criteria are patients aged 16 years or above; unresectable and/or metastatic STS; exacerbation within 6 months prior to registration; histopathological diagnosis of STS other than Ewing sarcoma, embryonal/alveolar rhabdomyosarcoma, well-differentiated liposarcoma and myxoid liposarcoma; prior doxorubicin-based chemotherapy for STS, and Eastern Cooperative Oncology Group performance status 0 to 2. The primary endpoint is progression-free survival, and the secondary endpoints include overall survival, disease-control rate, response rate, and adverse events. The total planned sample size to correctly select the most promising regimen with a probability of > 80% is 120. Thirty-seven institutions in Japan will participate at the start of this trial.

**Discussion:**

This is the first randomized trial to evaluate trabectedin, eribulin, and pazopanib as second-line therapies for advanced STS. We endeavor to perform a subsequent phase III trial comparing the best regimen selected by this study (JCOG1802) with GD.

**Trial registration:**

This study was registered with the Japan Registry of Clinical Trials (jRCTs031190152) on December 5, 2019.

## Background

Soft tissue sarcomas (STS) are rare malignancies that comprise a variety of histological diagnoses [1]. The standard treatment for STS is mainly based on the clinical stage and resectability of the tumor [2]. The standard treatment for resectable cases is surgical resection, whereas that for unresectable or metastatic advanced STS is chemotherapy.

Doxorubicin alone or in combination with ifosfamide or dacarbazine is widely accepted as first-line chemotherapy for advanced STS [2]. Doxorubicin induces irreversible cumulative cardiotoxicity, and second-line chemotherapy is required when the total dose approaches the upper limit or in tumors that are refractory to doxorubicin.

Trabectedin, eribulin, pazopanib, and gemcitabine plus docetaxel (GD) form the standard treatment options for second-line chemotherapy for advanced STS; however, there are no clear recommendations regarding the choice of regimen. The U.S. National Comprehensive Cancer Network guidelines list pazopanib, trabectedin, and eribulin as preferred regimens; other recommended regimens include dacarbazine, ifosfamide, temozolomide, vinorelbine, and regorafenib; pembrolizumab is also listed as a useful agent in specific cases [3]. The ESMO-EURACAN-GENTURIS guidelines recommend that second-line chemotherapy should be based on histopathology: trabectedin is a treatment option for all histological types, pazopanib should be used for non-liposarcoma, eribulin for liposarcoma only, and the GD and gemcitabine-dacarbazine combinations are preferred in patients who underwent prior treatment with doxorubicin-containing agents [2].

The results of clinical trials conducted to date {Table 1 [4–15]} indicate that trabectedin is highly effective for translocation-related sarcomas [6], liposarcoma and leiomyosarcoma (L-sarcoma) [4], eribulin for L-sarcoma, especially liposarcoma [9], pazopanib for other histological types besides liposarcoma[12], and GD for leiomyosarcoma [16], based on the histological inclusion criteria of each trial. These results have influenced the approval of each drug. While trabectedin is approved for L-sarcoma in the U.S.A., eribulin is approved exclusively for liposarcoma in the U.S.A and Europe, and pazopanib is approved for STS, excluding liposarcoma in the U.S.A and Europe. All three agents are approved for all histologic types of STS in Japan. Recent research, including that conducted using real-world data, has shown that all of these agents are effective against a wider range of histological types [17–19]. However, there is no clear evidence demonstrating the superiority of any one of these agents as second-line chemotherapy for advanced STS, since no randomized controlled trials have been conducted with these agents.

**Table 1:** Summary of median PFS and OS in clinical trials of trabectedin, eribulin, and pazopanib for advanced STS

Agent	**Histology**	**Study type**	**Year**	**n**	**Pre-treatment**	**PFS (months)**	**OS (months)**	**Ref**
Trabectedin	L-sarcoma	PhaseIII	2016	345	1 or more regimens incl. AI /2 or more regimens incl. anthracyclines	4.2	13.7	#4
Trabectedin	L-sarcoma	PhaseII	2009	136	1 or more regimens incl. anthracyclines or ifosfamide	3.3	13.9	#5
Trabectedin	TRS	PhaseII	2015	39	0 to 4 regimens	5.6	NR	#6
Trabectedin	STS	PhaseII	2005	104	1 or 2 regimens	3.5	9.2	#7
Trabectedin	STS	Retrospective	2015	885	-	4.4	12.2	#8
Eribulin	L-sarcoma	PhaseIII	2016	228	2 or more regimens incl. anthracyclines	2.6	13.5	#9
Eribulin	STS	PhaseII	2017	51	1 or more regimens incl. anthracyclines or ifosfamide	4.1	13.2	#10
Eribulin	Liposarcoma	PhaseII	2011	32	No more than 1 combination regimen /up to 2 single drug regimens	2.6	-	#11
Eribulin	Leiomyosarcoma	PhaseII	2011	38	No more than 1 combination regimen /up to 2 single drug regimens	2.9	-	#11
Eribulin	Synovial sarcoma	PhaseII	2011	19	No more than 1 combination regimen /up to 2 single drug regimens	2.6	-	#11
Eribulin	Others	PhaseII	2011	26	No more than 1 combination regimen /up to 2 single drug regimens	2.1	-	#11
Pazopanib	STS excluding liposarcoma	PhaseIII	2012	246	1 to 4 regimens incl. anthracyclines	4.6	12.5	#12
Pazopanib	STS	PhaseII	2009	142	0 to 2 regimes	3.0	10.6	#13
Pazopanib	Liposarcoma	PhaseII	2017	41	Any number of regimens	4.4	12.6	#14
Pazopanib	STS	Retrospective	2016	156	-	3.6	11.2	#15

*PFS* Progression-free survival, *OS* Overall survival, *Ref*. Reference, *L-sarcoma* Liposarcoma and leiomyosarcoma, *AI* Doxorubicin plus ifosfamide, *TRS* Translocation-related sarcoma, *NR* Not reached

Therefore, the Bone and Soft Tissue Tumor Study Group (BSTTSG) of the Japan Clinical Oncology Group (JCOG) planned to establish a standard second-line treatment for advanced STS from among the widely used regimens such as trabectedin, eribulin, pazopanib, and GD. First, we plan to conduct a selection design, randomized, phase II study using trabectedin, eribulin, and pazopanib, which are relatively new drugs for STS that have been approved since the 2010s. A randomized phase III study comparing the best second-line agent determined by this JCOG1802 study with GD, which has been in use since the 2000s and is currently deemed standard therapy for STS, will be planned.

## Methods/design

### Study design

The objective of this clinical trial (JCOG1802, 2ND-STEP) is to determine the most promising regimen among trabectedin, eribulin, and pazopanib, which will be designated as the test arm in a future phase III trial of second-line treatment for patients with metastatic or unresectable STS that has progressed after first-line chemotherapy with doxorubicin.

This multicenter, randomized, open-label, parallel-arm, selection design phase II trial aims to examine the efficacy and safety of trabectedin, eribulin, and pazopanib, which are widely used anticancer agents for advanced STS.

At commencement, this study will be conducted across 37 institutions in Japan, all of which are participants in the BSTTSG of the JCOG. Patients with advanced STS are being treated at all participating institutions, and potentially eligible patients will be registered by investigators. The study protocol was approved by the National Cancer Center Hospital Certified Review Board for Clinical Trials (CRB Certification No. CRB3180008) prior to the initiation of patient accrual.

The inclusion and exclusion criteria for this study are summarized in Table 2.

**Table 2:** Inclusion and exclusion criteria of JCOG1802

Inclusion criteria	**Exclusion criteria**
(1) Histologically-proven STS (2) H&E slides used for diagnosis of STS are available (3) STS with metastatic disease (Stage IV in AJCC- TNM 8th edition), unresectable primary sarcoma, or unresectable recurrent sarcoma (4) Disease progression confirmed within 6 months (5) Disease to fulfill either following (i)-(iv), chemotherapy here including perioperative chemotherapy (i) Disease progression during doxorubicin-based chemotherapy (ii) Disease progression after termination of doxorubicin-based chemotherapy because of other than disease progression during chemotherapy (iii) Local recurrence and/or metastasis of disease which has been disappeared by treatment including doxorubicin-based chemotherapy (iv) Disease progression of residual disease after doxorubicin-based chemotherapy (6) History of chemotherapy for sarcoma is only doxorubicin-based chemotherapy (7) No treatment history for sarcoma and other cancers with trabectedin, eribulin or pazopanib (8) Age 16 years or older (9) ECOG (Eastern Cooperative Oncology Group) performance status (PS): 0–2 (10) Measurable or non-measurable disease (11) Oral intake possible (12) Adequate organ function as proved by the following laboratory studies within 14 days prior to registration; neutrophil count ≥ 1,500 /mm3; hemoglobin ≥ 8.0 g/dL; platelet count ≥ 100,000 /mm3; TB (total bilirubin) ≤ 1.5 mg/dL; AST ≤ 100 U/L; ALT ≤ 100 U/L; renal function to fulfill either following (a) or (b); (a) creatinine ≤ 1.5 mg/dL; (b) creatinine clearance ≥ 50 mL/min; ALP ≤ 900 U/L; CPK ≤ 600 U/L for men, ≤ 400 U/L for women; PT-INR ≤ 1.38; APTT ≤ 44.4 s; TSH: 0.5–4.5 micro unit/mL; FT4: 0.9–1.8 ng/dL (13) Normal findings or no need of treatment on electrocardiogram (ECG) within 28 days prior to registration (14) More than 50% of ejection fraction in echocardiography within 28 days prior to registration (15) Written informed consent. If the patient's age is under 18 years old, the additional consent must be obtained from the patient's parent or legal guardian	(1) Synchronous or metachronous (within 2 years) malignancy except cancer with 95% or more of 2-year relative survival rate (2) Diagnosed with active infectious disease requiring systemic treatment (3) Febrile more than 38 degrees Celsius at the registration (4) Pregnancy, possible pregnancy, within 28 days after delivery or breast-feeding, or male subjects who want pregnancy of their partner (5) Psychological disorder difficult to participate in this study (6) Receiving continuous systemic corticosteroid or immunosuppressant treatment (PO or IV) (7) Systolic blood pressure > 140 mmHg and diastolic blood pressure > 90 mmHg in two consecutive times measured in a hospital on the day of registration (irrespective of antihypertensive agents use) (8) Interstitial pneumonia, pulmonary fibrosis or severe emphysema confirmed on chest CT (9) HBs antigen positive (10) History of cardiovascular disease as follow; (a) Myocardial infarction within the last 6 months, unstable angina pectoris within the last 3 weeks (b) Angioplasty surgery or stent implantation, or coronary artery bypass graft within 6 months (c) Heart failure class III or IV according to NYHA Classification (d) Uncontrolled valve disease, dilative cardiomyopathy, or hypertrophic myocardiopathy (11) Active bleeding (12) Symptomatic brain metastasis or brain metastasis requiring treatment (screening of brain metastasis is not required for clinically unnecessary patients) (13) Pleural effusion, ascitic fluid, pericardial effusion requiring drainage

**Fig. 1:**
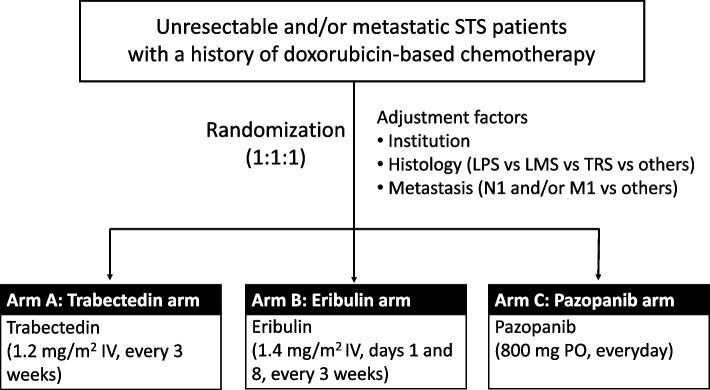
Flowchart of JCOG1802 (2ND-STEP) study. STS: Soft tissue sarcoma; LPS: Liposarcoma; LMS: Leiomyosarcoma; TRS: Translocation-related sarcoma; IV: Intravenous drip; PO: Per os (Oral administration)

After confirming participant eligibility, patients will be randomly assigned to the treatment arms at the JCOG Data Center. Random allocation to trabectedin, eribulin, and pazopanib will be performed using a minimization method in a ratio of 1:1:1. The institution, histology (liposarcoma vs. leiomyosarcoma vs. translocation-related sarcoma vs. other), and distant metastases (N1 and/or M1 vs. other) will serve as factors for allocation adjustment (Fig. 1).

### Interventions

Patients will be randomized to arms A, B, or C (Fig. 1) as follows. arm A, intravenous drip infusion of trabectedin 1.2 mg/m^2^ (body surface area) on day 1 for 24 h every 3 weeks; arm B, intravenous drip infusion of eribulin 1.4 mg/m^2^ (of body surface area) on days 1 and 8 for 2–5 min every 3 weeks; and arm C, oral administration of pazopanib 800 mg/day more than 1 h before meals or more than 2 h after meals every day.

The criteria for dose reduction for each arm are set as follows. There are three dose levels of trabectedin in arm A: level 0 (full dose), 1.2 mg/m^2^; level 1, 1.0 mg/m^2^; and level 2, 0.8 mg/m^2^. There are three dose levels for eribulin in arm B: level 0 (full dose), 1.4 mg/m^2^; level 1, 1.1 mg/m^2^; and level 2, 0.7 mg/m^2^. There are four dose levels for pazopanib in arm C: level 0 (full dose), 800 mg/body; level 1, 600 mg/body; level 2, 400 mg/body; and level 3, 200 mg/body. The dose will be lowered by one level in the next course, in the event of severe myelosuppression, liver dysfunction, or cardiac dysfunction. The treatment protocol will be terminated if any toxicity is observed even at the lowest dose level.

The concomitant use of any of the following therapies is prohibited during administration of the treatment protocol: (1) anticancer drugs other than the treatment regimen, (2) radiation therapy (including particle therapy) for the target lesion, and (3) immunotherapy.

### Participant timeline

The participant timeline is summarized in Table 3. Candidates who consent to participate will be checked for eligibility based on the inclusion/exclusion criteria, and enrollment will be completed after confirmation of participant eligibility. Physical examination, Eastern Cooperative Oncology Group performance status (PS), body weight, body height, complete blood count (CBC), serum biochemistry, activated partial prothrombin time, prothrombin time-international normalized ratio, thyroid stimulating hormone, free T4, and urine analyses are performed within 14 days before registration. Echocardiography, electrocardiography, contrast-enhanced computed tomography (CT) of the chest, abdomen, and pelvis, and additional CT/magnetic resonance imaging (MRI) of the lesions will be performed within 28 days before registration. Plain CT is acceptable when contrast agents cannot be administered due to allergies, asthma, etc.

**Table 3:**
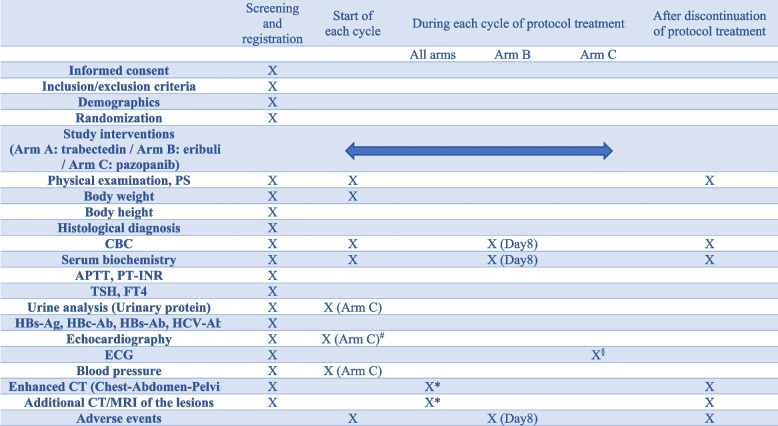
The study schedule

^#^Echocardiography is performed in Arm C if any cardiac-related symptoms are observed.^§^ECG will be performed on Day 28 of Arm C and every 8 weeks thereafter.^*^Enhanced CT of chest-abdomen-pelvis, and additional CT/MRI of the lesions to determine efficacy will be performed every 4 weeks on the first four occasions after initiation of protocol treatment, and every 6 weeks thereafter

Participants will commence the treatment protocol within 14 days of registration. The treatment protocol is continued until one of the following termination criteria are met: (1) exacerbation of disease (judged as ineffective treatment); (2) the treatment protocol cannot be continued due to adverse events including grade 4 non-hematologic toxicity and delay of 56 days before the start of the next course; (3) the patient requests termination of treatment for reasons related to adverse events; (4) the patient requests termination of treatment for reasons unrelated to adverse events; (5) death during treatment; and (6) post-enrollment exacerbation prior to the initiation of treatment (inability to start the treatment protocol due to rapid progression), discovery of protocol violation, change of treatment due to a change in the pathological diagnosis after enrollment, or other reasons that make the patient ineligible for treatment.

Treatment efficacy will be determined by performing contrast-enhanced chest-abdomen-pelvis CT and additional CT/MRI of the lesions every 4 weeks on the first four occasions after initiation of the treatment protocol, and every 6 weeks thereafter. After termination of the treatment protocol, physical examination, PS, body weight, CBC, serum biochemistry, and adverse events will be assessed every 6 months. If treatment is terminated for reasons other than progression of the disease, 6-weekly CT examination will be continued until disease progression.

### Primary endpoint

The primary endpoint is progression-free survival (PFS). PFS is defined as the period from the date of registration until the date of progression or the date of death from any cause, whichever is earlier. Herein, progression includes both progressive disease as determined by imaging based on the revised Response Evaluation Criteria in Solid Tumours (RECIST) guidelines, and clinical progression without confirmation by imaging studies.

### Secondary endpoints

The secondary endpoints include overall survival, disease-control rate, response rate, and proportion of adverse events (adverse reactions). Overall survival is defined as the period from the date of registration to the date of death from any cause. The disease-control rate is defined as the proportion of patients with either complete response (CR), partial response (PR), or stable disease according to the RECIST criteria from amongst all eligible patients with measurable disease. The response rate is defined as the proportion of patients whose best overall response is either CR or PR amongst all eligible patients with measurable disease. The proportion of adverse events (adverse reactions) is defined as the frequency of adverse events (toxicity) of the worst severity according to the Common Terminology Criteria for Adverse Events v5.0-JCOG during the entire course.

### Sample size calculation

The required sample size to accurately ascertain the most favorable treatment based on the point estimate of the hazard ratio (HR) was calculated using Monte Carlo simulation corresponding to the instance where an exponential distribution is assumed for Liu’s selection design [20]. Based on previous studies [4–15], we established an accrual period of 3 years, follow-up period of 6 months, and the median PFS for each arm to be 2, 2, and 4 months in condition 1; 2, 3, and 4 months in condition 2; and 3, 3, and 4 months in condition 3. Under these conditions, the required sample size to correctly select the most favorable treatment arm with a probability of 80% was 7 for condition 1, 20 for condition 2, and 34 for condition 3. To maintain the probability of correct selection of at least 80% in any condition, the planned sample size was set at 40 for each arm and the total sample size was 120, where the probability was 99.7% for condition 1, 88.8% for condition 2, and 83.0% for condition 3.

### Data collection, management, monitoring, and auditing

Data entry into the electronic case report form is performed by investigators using an electronic data capture (EDC) system via the JCOG Web Entry System. Adverse events are closely monitored by investigators. Investigators will report severe adverse events to the institutional administrator and principal investigator, and then to the CRB of the National Cancer Center Hospital, as appropriate.

Clinical data entry, data management, and central monitoring will be performed using the EDC system, E-DMS Online (EPS Corporation, Tokyo, Japan). All statistical analyses will be conducted at the JCOG Data Center. Interim analysis is not planned for this study because the follow-up period for the primary analysis is short and any safety issues with participants can be ascertained through periodic monitoring. In-house monitoring will be performed every 6 months by the JCOG Data Center to evaluate the study progress and improve the quality of the data.

### Statistical analysis

Statistical analysis will be performed at the JCOG Data Center. The primary analysis will be performed 6 months after the end of accrual, when collection of the primary endpoint data for all enrolled patients is expected to be complete. The treatment protocol with the best point estimate of the HR for PFS, which is the primary endpoint of this study, will be the test treatment arm in a subsequent phase III trial. However, the treatment arm for the phase III trial will be decided comprehensively if the following results are obtained, taking into consideration endpoints other than PFS. First, the PFS obtained is substantially lower than expected (insufficient results for promising therapy). Second, the overall survival results differ significantly from those of PFS. Third, the frequency of adverse events among the arms differs significantly from the expected frequency.

For the primary analysis of PFS, the respective HRs of arms A to B and A to C will be calculated using an unstratified Cox proportional-hazards model for all enrolled patients, and the treatment with the best HR will be judged to be the most promising regimen. Since this study does not make judgments based on hypothesis testing, no significance level is set a priori, and no adjustment will be made for multiplicity.

Subgroup analyses based on the factors mentioned below are to be conducted, as necessary. The factors for which subgroup analyses are planned include age group 1 (< 40/ ≥ 40 years), age group 2 (< 70/ ≥ 70 years), sex (male/female), PS (0/1 and 2), histological type (liposarcoma/leiomyosarcoma/translocation-related sarcoma/other), distant metastasis 1 [(M1 and/or N1)/other], distant metastasis 2 [M1/(N1 and M0)/other], and doxorubicin (perioperative chemotherapy/palliative chemotherapy).

## Discussion

Most guidelines state that first-line chemotherapy for advanced STS should include doxorubicin alone or in combination with ifosfamide or dacarbazine; however, there are no clear guidelines for standard second-line therapy. One of the chief factors preventing the establishment of clear drug selection criteria for second-line chemotherapy for advanced STS is the lack of clinical trials that directly compare the efficacy and safety of drugs used for this purpose. Therefore, data from clinical trials with different types of participants are used as a reference for drug selection based on indirect comparison. We intend to overcome this problem by conducting this multicenter, randomized, open-label, parallel-arm, selection design phase II trial of the three major treatment options for second-line chemotherapy for advanced STS, viz. trabectedin, eribulin, and pazopanib. The JCOG 1802 trial (a randomized phase II trial of trabectedin, eribulin, and pazopanib as second-line treatment for advanced soft-tissue sarcoma after doxorubicin, which is also known as the 2ND-STEP trial) will be the first randomized trial using trabectedin, eribulin, and pazopanib for STS worldwide. This trial is expected to determine the most promising regimen from amongst trabectedin, eribulin, and pazopanib as the test arm regimen in a future phase III trial of second-line treatment for advanced STS patients.

## Data Availability

Not applicable.

## Abbreviations

GD: Gemcitabine plus docetaxel
L-sarcoma: Liposarcoma and leiomyosarcoma
STS: Soft tissue sarcoma
JCOG: Japan Clinical Oncology Group
BSTTSG: Bone and Soft Tissue Tumor Study Group
PFS: Progression-free survival
RECIST: Response Evaluation Criteria in Solid Tumours
EDC: Electronic data capture
CR: Complete response
PR: Partial response
HR: Hazard ratio
PS: Performance status
CBC: Complete blood count
CT: Computed tomography
MRI: Magnetic resonance imaging
